# The Effect of COVID-19 Vaccines on Chronic Inflammatory Remodeling in NSTEMI Patients: A Galectin-3-Based Single-Center Study

**DOI:** 10.3390/jcm15135312

**Published:** 2026-07-07

**Authors:** Adem Koksal, Mesut Tomakin, Mehmet Seyfettin Saribaş, Fatih Akkaya, Fatmanur Cavdaroglu Ustabas, Ibrahim Caltekin, Diler Us Altay, Tevfik Noyan, Ali Aygun

**Affiliations:** 1 Department of Emergency Medicine, Faculty of Medicine, Ordu University, Ordu 52200, Türkiye; drademkoksal@gmail.com (A.K.); m.seyfettinsaribas@odu.edu.tr (M.S.S.); fatmanurcavdaroglu96@gmail.com (F.C.U.); drcaltekin@gmail.com (I.C.); dr_aliaygun@hotmail.com (A.A.); 2 Department of Emergency Medicine, Turhal State Hospital, Tokat 60300, Türkiye; 3 Department of Cardiology, Faculty of Medicine, Ordu University, Ordu 52200, Türkiye; drfatihakkaya@gmail.com; 4 Department of Nutrition and Dietetics, Faculty of Health Sciences, Ordu University, Ordu 52200, Türkiye; surelid@hotmail.com; 5 Department of Medical Biochemistry, Faculty of Medicine, Ordu University, Ordu 52200, Türkiye; tevfiknoyan@hotmail.com

**Keywords:** Acute Coronary Syndrome (ACS), NSTEMI, Galectin-3, COVID-19 vaccine, inflammation

## Abstract

**Background:** This study evaluated the potential effects of different COVID-19 vaccine platforms (mRNA and inactivated) on acute coronary syndrome (ACS) through Galectin-3, a biomarker of chronic inflammation and fibrosis. It aimed to compare serum Galectin-3 levels among NSTEMI patients according to COVID-19 vaccination status and vaccine type. **Methods:** A total of 75 patients with NSTEMI were prospectively enrolled and categorized into three groups: inactivated vaccine recipients (n = 25), mRNA vaccine recipients (n = 25), and unvaccinated controls (n = 25). Serum Galectin-3 levels were measured to assess chronic inflammatory status. Additionally, markers of acute myocardial injury and inflammatory response were analyzed. **Results:** Galectin-3 levels were similar across the inactivated vaccine, mRNA vaccine, and unvaccinated NSTEMI group, with no statistically significant difference observed (*p* = 0.481). Although troponin I levels and acute inflammatory cell burden were higher in vaccinated patients compared with the unvaccinated NSTEMI group, Galectin-3 levels remained comparable among all groups. No significant differences in Galectin-3 levels were observed according to vaccination status or vaccine type. **Conclusions:** In this exploratory cohort of NSTEMI patients, serum Galectin-3 levels did not differ significantly according to COVID-19 vaccination status or vaccine type. These findings suggest no detectable association between vaccination history and Galectin-3 levels in the study population. Larger prospective studies with longitudinal follow-up are needed to confirm these observations.

## 1. Introduction

Acute coronary syndromes (ACS) continue to be one of the leading causes of global mortality and morbidity. The pathogenesis of this condition is fundamentally based on complex inflammatory pathways, endothelial dysfunction, and plaque instability [1]. The ongoing COVID-19 pandemic has established a new paradigm in clinical cardiology as a potent cardiovascular stressor that triggers systemic hyperinflammation and prothrombotic states [2]. Following the rapid global implementation of various vaccine platforms, the effects of vaccination—particularly in patients with a history of ACS—have drawn considerable attention [3,4,5]. In this context, understanding the long-term effects of vaccination on cardiac remodeling and inflammatory status has become a clinical priority for the international medical community.

In Europe, more than 75% of the population has completed the primary vaccination schedule, predominantly using vaccines produced by mRNA and viral vector-based vaccine technologies [6]. Although the European Society of Cardiology (ESC) and the European Medicines Agency (EMA) have consistently confirmed the high safety profile of these vaccines, rare cardiovascular adverse events such as myocarditis and pericarditis have been reported, particularly in younger populations [7,8]. However, a large proportion of the existing European literature focuses on mRNA-based vaccines (BNT162b2). This situation creates a significant knowledge gap regarding the comparative cardiovascular effects of inactivated virus platforms (such as CoronaVac), which are widely used in Eastern Europe, Asia, and the Middle East [9]. Nevertheless, there are a limited number of studies evaluating the subacute and chronic cardiac effects of different COVID-19 vaccine technologies (mRNA, viral vector, etc.) [10,11], and further research in this area is still needed.

Galectin-3, a beta-galactoside-binding lectin, has emerged as a critical biomarker in this field of research due to its role in mediating myocardial fibrosis, chronic inflammation, and ventricular remodeling processes [12]. Galectin-3 is a biomarker associated with inflammation, fibrosis, and tissue remodeling. However, its utility in detecting potential long-term biological effects related to vaccination remains uncertain and should be considered exploratory. Unlike traditional necrosis markers such as troponin, the temporal kinetics of Galectin-3 provide unique insights into persistent inflammatory burden and the long-term risk of heart failure following ACS [13]. Recent studies conducted in European cohorts have demonstrated that Galectin-3 is a strong predictive biomarker for adverse outcomes (severe disease, need for intensive care, mortality) in patients with COVID-19 [14,15,16,17]. Galectin-3 levels are used to predict disease severity and poor prognosis, particularly in relation to processes involving inflammation, fibrosis, and thrombosis [14,15,16,17,18]. During an average follow-up period of approximately 3 years (2.8–3.5 years) after acute myocardial infarction, the risk of recurrent major cardiovascular events or death was increased by 3–9-fold in patients with Galectin-3 levels > 8.7–9.2 ng/mL [19,20].

Considering the heterogeneity in global vaccination strategies, there is a need for prospective data evaluating whether different vaccine types differentially affect cardiac recovery and remodeling processes. This study aims to compare serum Galectin-3 levels among NSTEMI patients according to COVID-19 vaccination status and vaccine type and to explore possible associations between vaccination history and biomarkers related to inflammatory remodeling.

## 2. Materials and Methods

This study was conducted between 1 January 2025, and 30 November 2025, and was initiated after obtaining approval from the Ordu University Non-Interventional Clinical Research Ethics Committee (decision no: 2024/198). The study was designed as an observational cross-sectional study in which eligible patients were prospectively enrolled during the study period. Given the observational cross-sectional design, the findings were intended to evaluate associations between COVID-19 vaccination status and serum Galectin-3 levels rather than establish causal relationships.

### 2.1. Study Population

Patients who presented to the emergency department, were diagnosed with NSTEMI, were willing to participate in the study, were over 18 years of age, and whose vaccination information was accessible through the national personal health system were included in the study. Diagnoses were established based on relevant clinical, electrocardiographic findings, laboratory findings, and angiography reports. At the time of presentation to the emergency department, patients’ age, sex, comorbid chronic diseases, history of prior COVID-19 infection, and COVID-19 vaccination status were recorded in the data collection form prepared for the study. In addition, along with the presenting complaint, vital parameters such as body temperature, heart rate, peripheral oxygen saturation (SpO_2_), systolic blood pressure (SBP), and diastolic blood pressure (DBP) were recorded. For clarity, the comparison group consisted of unvaccinated patients with NSTEMI rather than healthy controls; therefore, all analyses were performed within an ACS population stratified according to vaccination history.

### 2.2. Exclusion Criteria

Patients with the following characteristics were excluded from the study: acute cerebrovascular events, acute/chronic renal failure, acute decompensated heart failure, concomitant additional ischemic pathologies, use of ACE inhibitors, use of statin group medications (These medications were included among the exclusion criteria because previous studies have suggested that statins and renin–angiotensin system inhibitors may influence Galectin-3 expression and circulating levels. Their exclusion was intended to reduce potential pharmacological confounding in the assessment of Galectin-3 concentrations [21,22,23]), sepsis, severe anemia, oncological diseases, autoimmune diseases, pregnancy, and receipt of two or more different COVID-19 vaccines.

In addition, it is known that Galectin-3 is particularly more effective in reflecting chronic inflammation (over 3 years) [19,20]. Individuals whose most recent COVID-19 vaccination was administered after 1 January 2022 were excluded to minimize the potential influence of short-term post-vaccination immune responses and to focus the analysis on individuals with more remote vaccination histories. Furthermore, patients with incomplete data were also excluded from the study.

### 2.3. Laboratory Analyses

Routine blood tests were obtained from the patients at the time of presentation. In addition to routine samples, additional venous blood samples were collected for biochemical, hemogram, and coagulation analyses (citrate-containing, blue-top tubes). From the obtained samples, biochemical parameters (glucose, creatinine, sodium, and potassium), hematological parameters (white blood cells [WBC], neutrophils, lymphocytes, immature granulocytes [IG Count]), coagulation parameters (fibrinogen and D-dimer), and cardiac and inflammatory markers (troponin and Galectin-3) were analyzed.

### 2.4. Galectin-3 Measurement

Blood samples obtained during routine evaluation at baseline were used, and no additional blood was collected for this measurement. From each individual, 5 mL of venous blood was drawn into 13 × 100 mm Vacutainer^®^ tubes for routine biochemical tests, and the samples were allowed to stand at room temperature for approximately 30 min. Subsequently, the samples were stored at 4 °C and centrifuged at 3000 rpm for 10 min to separate the serum. The obtained serum samples were transferred into Eppendorf tubes and stored at −80 °C until the time of analysis. Serum Galectin-3 levels were measured using a human Galectin-3 enzyme-linked immunosorbent assay (ELISA) kit (Bioassay Technology Laboratory, Shanghai, China, Cat. No. E1951Hu) in accordance with the manufacturer’s instructions. Absorbance values were read at a wavelength of 450 nm using a BioTek Instruments EL800 Microplate Reader (Agilent Technologies, Inc., Winooski, VT, USA). The analytical standard curve range of the ELISA kit was 5–2000 pg/mL, and the limit of detection (sensitivity) was 2.49 pg/mL. According to the manufacturer’s strict instructions, no dilution was applied to the samples to avoid sample matrix interference. The raw results obtained from the microplate reader were initially calculated in pg/mL and subsequently converted to ng/mL (by dividing by 1000) to ensure comparability with the prognostic cut-off points established in the cardiovascular literature.

### 2.5. Angiographic Evaluation

All patients were evaluated by the cardiology department and underwent coronary angiography. Patients in whom critical coronary stenosis was detected as a result of the angiographic examination and who underwent percutaneous coronary intervention were included in the study.

### 2.6. Sample Size and Grouping

A priori sample size analysis was performed using G*Power software (Version 3.1.9.7). The primary analysis was defined as the comparison of serum Galectin-3 levels among the inactivated vaccine, mRNA vaccine, and unvaccinated NSTEMI groups. Since no directly comparable study based on this specific patient population and biomarker was available in the literature, the effect size was assumed to be Cohen’s f = 0.40. For a one-way ANOVA model, assuming α = 0.05, a statistical power of 0.80, and three groups, the minimum required sample size was calculated as 22 patients per group, yielding a total sample size of 66 patients. A total of 75 patients were included in the study, with 25 patients allocated to each group. Patients were divided into three groups according to their COVID-19 vaccination status:

Group A: Inactivated vaccine group; patients who received PiCoVacc/CoronaVac
Group B: mRNA vaccine group; patients who received the BNT162b2 vaccine
Group C: Unvaccinated NSTEMI group; patients who had not received any COVID-19 vaccine

Each group was planned to consist of 25 patients. During the study period, once the targeted number of patients for each group was reached, further patient recruitment for the respective group was stopped; after completion of all groups, the analysis phase was initiated. Equal group sizes were intentionally targeted to facilitate balanced comparisons of Galectin-3 levels across vaccination categories. However, this sampling strategy was not intended to reflect the natural distribution of vaccination status among patients with NSTEMI.

### 2.7. Statistical Analyses

Statistical analyses were performed using IBM SPSS Statistics for Windows, Version 30.0 (IBM Corp., Armonk, NY, USA). The distribution of continuous variables was evaluated using visual methods, including histograms and Q-Q plots, and analytical normality tests. Normally distributed continuous variables were expressed as mean ± standard deviation, whereas non-normally distributed variables were presented as median with interquartile range (Q1–Q3).

Comparisons among the Sinovac, BioNTech, and unvaccinated control groups were performed using one-way analysis of variance (ANOVA) for normally distributed variables and the Kruskal–Wallis test for non-normally distributed variables. For variables with a statistically significant Kruskal–Wallis result, pairwise post hoc comparisons were conducted using the Dunn–Bonferroni method, and Bonferroni-adjusted *p* values were reported to account for multiple comparisons.

Comparisons according to the history of COVID-19 were performed using the independent samples *t*-test for normally distributed continuous variables and the Mann–Whitney U test for non-normally distributed variables.

To address potential confounding due to baseline imbalances among the study groups, additional general linear models were constructed using natural log-transformed Galectin-3 [Ln(Galectin-3)] as the dependent variable. The vaccination group was entered as the main independent variable, with the unvaccinated NSTEMI group used as the reference category. Three sequential models were evaluated. Model 1 included the vaccination group only. Model 2 was adjusted for age, sex, and previous COVID-19 history. Model 3 was additionally adjusted for systolic blood pressure, body temperature, white blood cell count, glucose, troponin, fibrinogen, and D-dimer. Estimated marginal means were calculated for vaccination groups, and Bonferroni correction was applied for pairwise comparisons. Homogeneity of error variances was evaluated using Levene’s test.

To further evaluate the potential influence of previous COVID-19 infection, a sensitivity analysis was performed after excluding patients with a history of COVID-19 infection. In this restricted complete-case cohort, the same sequential modeling strategy was applied, except that previous COVID-19 history was not included as a covariate. Accordingly, Model 1 included the vaccination group only, Model 2 was adjusted for age and sex, and Model 3 was additionally adjusted for systolic blood pressure, body temperature, white blood cell count, glucose, troponin, fibrinogen, and D-dimer.

All statistical tests were two-tailed, and a *p* value of <0.05 was considered statistically significant.

## 3. Results

### 3.1. Baseline Characteristics of Participants

A total of 75 participants were included in the study; 25 of them were in Group A, 25 in Group B, and 25 in Group C (unvaccinated NSTEMI group). No statistically significant difference was found between the groups in terms of age (*p* = 0.556). The mean age was 65.72 ± 13.82 years in Group A, 64.80 ± 10.71 years in Group B, and 62.92 ± 6.10 years in Group C.

SBP showed a statistically significant difference between the groups (*p* = 0.022). The median SBP value was 130 (115–140) mmHg in Group A, 120 (115–155) mmHg in Group B, and 120 (110–120) mmHg in Group C. A significant difference was also observed between the groups in terms of DBP (*p* = 0.021). No statistically significant difference was found between the groups regarding heart rate (*p* = 0.653). Body temperature was 36.6 (36.2–36.7) °C in Group A, 36.5 (36.0–36.6) °C in Group B, and 36.2 (36.0–36.45) °C in Group C, and the difference between the groups was statistically significant (*p* = 0.006). The findings are summarized in Table 1.

### 3.2. Laboratory Findings

White blood cell (WBC) counts showed a statistically significant difference between the groups (*p* = 0.003). The median WBC value was 10.87 (6.93–14.15) ×10^9^/L in Group A, 10.30 (8.27–12.20) ×10^9^/L in Group B, and 7.60 (6.16–8.51) ×10^9^/L in Group C. Similarly, neutrophil counts were significantly higher in the vaccinated groups compared to the unvaccinated NSTEMI group (*p* = 0.001). No statistically significant difference was observed between the groups in terms of lymphocyte counts (*p* = 0.295).

Blood glucose levels were markedly higher in Groups A and B compared to the unvaccinated NSTEMI group (*p* < 0.001). The median glucose value was 142 (115–211.5) mg/dL in Group A, 149 (120–228.5) mg/dL in Group B, and 94 (88.5–116.5) mg/dL in Group C. IgG levels were also found to be significantly higher in the vaccinated groups compared to the unvaccinated NSTEMI group (*p* = 0.002).

Troponin I levels showed a statistically significant difference between the groups (*p* < 0.001). The median troponin value was 1.0 (0.43–2.9) ng/mL in Group A, 2.6 (0.62–9.43) ng/mL in Group B, and 0.1 (0.1–0.72) ng/mL in Group C. Similarly, D-dimer levels were significantly higher in the vaccinated groups compared to the unvaccinated NSTEMI group (*p* = 0.002). A statistically significant difference was also observed between the groups in terms of fibrinogen levels (*p* = 0.048).

No statistically significant difference was observed between the groups in terms of sodium and potassium levels (*p* = 0.591 and *p* = 0.629, respectively). Galectin-3 levels were similar across all groups and did not reach statistical significance (*p* = 0.481). The findings are summarized in Table 1.

### 3.3. Subgroup Analysis According to COVID-19 History

Among the participants, 55 had no history of COVID-19, while 20 had a previous history of COVID-19. No statistically significant difference was found between the two groups in terms of age, SBP, DBP, heart rate, WBC, neutrophil, lymphocyte, glucose, IgG, troponin, fibrinogen, D-dimer, sodium, and potassium levels (all *p* > 0.05).

Body temperature was significantly higher in the group with a history of COVID-19 (*p* = 0.035). Although Galectin-3 levels were higher in the group with a history of COVID-19, this difference was not statistically significant (*p* = 0.320). The findings are summarized in Table 2.

Post hoc Dunn–Bonferroni analyses showed that WBC, neutrophil count, glucose, immature granulocyte count and troponin levels were significantly higher in both vaccinated groups compared with Group C (*p* < 0.05). However, no significant differences were observed between Group A and Group B for these parameters (*p* > 0.05). D-dimer levels differed significantly only between Group C and Group A (*p* < 0.05). Importantly, pairwise comparisons revealed no significant difference in Galectin-3 levels among Group C, Group A and Group B (*p* > 0.05). The findings are summarized in Table 3.

In the overall cohort, three sequential general linear models were constructed to evaluate the association between vaccination group and log-transformed Galectin-3 levels. In the crude model, vaccination group was not significantly associated with Ln(Galectin-3) levels (F = 0.072, *p* = 0.931). This finding remained unchanged after adjustment for age, sex, and previous COVID-19 history (F = 0.053, *p* = 0.949). In the fully adjusted model, which additionally included systolic blood pressure, body temperature, WBC count, glucose, troponin, fibrinogen, and D-dimer, vaccination group remained non-significantly associated with Ln(Galectin-3) levels (F = 0.583, *p* = 0.562). Compared with the unvaccinated NSTEMI group, neither the inactivated vaccine group (B = 0.270, 95% CI: −0.260 to 0.800, *p* = 0.313) nor the mRNA vaccine group (B = 0.247, 95% CI: −0.272 to 0.765, *p* = 0.345) showed a statistically significant difference in the fully adjusted model. Bonferroni-adjusted pairwise comparisons based on estimated marginal means were also non-significant across all models. The findings are shown in Table 4 and Table 5.

A sensitivity analysis was then performed after excluding patients with previous COVID-19 infection. In this restricted cohort, vaccination group was not significantly associated with Ln(Galectin-3) levels in the crude model (F = 1.315, *p* = 0.277), the age- and sex-adjusted model (F = 1.390, *p* = 0.259), or the fully adjusted model (F = 1.310, *p* = 0.280). In the fully adjusted sensitivity model, neither the inactivated vaccine group (B = 0.020, 95% CI: −0.502 to 0.541, *p* = 0.940) nor the mRNA vaccine group (B = 0.333, 95% CI: −0.181 to 0.847, *p* = 0.198) differed significantly from the unvaccinated NSTEMI group. The findings are shown in Table 6 and Table 7.

## 4. Discussion

COVID-19 infection has brought one of the most debated topics regarding whether vaccines increase cardiovascular risks. When the current literature is reviewed, Botton et al. reported that mRNA vaccines are not associated with serious cardiovascular events such as ACS, pulmonary embolism, and stroke, and that the limited increased risk observed with viral vector-based vaccines remains lower compared to the risks caused by COVID-19 infection. In the same study, it was also stated that a transient increase in the risk of myocardial infarction and pulmonary embolism may occur in the second week following the first dose of viral vector-based vaccines [24]. Some studies in the literature emphasize that ACS is already a common clinical condition in the general population and that a significant proportion of post-vaccination cases may be coincidental. In addition, proposed mechanisms for post-vaccination myocardial infarction cases, such as stress, Kounis syndrome, and rare coagulation disorders, are stated not to imply that vaccines directly cause coronary occlusion [25]. Huang et al. reported that mRNA vaccines are more commonly associated with myocarditis, although this risk is very rare [26]. When all these findings are evaluated together, it is concluded that although rare cardiovascular adverse events may be observed after vaccination, the clear clinical benefit of vaccination becomes evident when considering the severe disease, mortality, and cardiovascular complications that may be caused by COVID-19. It is generally accepted that the cardiovascular risk caused by COVID-19 infection is higher and more severe than the potential adverse effects of vaccines. In the study conducted by Nahab et al., it was reported that having concurrent COVID-19 infection within the first 21 days after vaccination increased the risk of ischemic and hemorrhagic stroke approximately 8-fold, regardless of vaccine type [27]. Similarly, in the study conducted by Tu et al., it was shown that the risk of cerebral venous thrombosis after COVID-19 infection was 32 times higher in unvaccinated individuals compared to those vaccinated with mRNA vaccines [28]. Importantly, because all participants in the present study had already experienced NSTEMI, the study design does not permit assessment of vaccine-related risk, predisposition to ACS, incidence of cardiovascular events, or overall cardiovascular safety. Rather, the findings are limited to comparisons of Galectin-3 levels within an NSTEMI population stratified according to vaccination history. Accordingly, our findings should be considered exploratory and descriptive, serving primarily to generate hypotheses for future prospective studies rather than to establish causal associations.

It is known that in NSTEMI cases, the increase in troponin levels is generally limited, whereas in STEMI cases, higher values are reached [29,30]. In addition, it has previously been reported that immune activation following mRNA vaccination may have effects on endothelial function and the microvascular circulation [31,32]. Previous studies have proposed that immune responses triggered by vaccination may influence inflammatory pathways and endothelial function; however, whether such mechanisms contribute to differences in biomarker levels during acute NSTEMI remains uncertain [33]. The observed differences in troponin levels should be interpreted cautiously, as they may reflect variation in clinical presentation and NSTEMI severity rather than vaccination-related effects. In our study, higher troponin levels were observed in the mRNA-vaccinated group; however, the observational design of the study does not allow determination of whether this finding is related to vaccination status or to differences in infarct severity, timing of presentation, or other clinical characteristics.

Higher D-dimer and fibrinogen levels were observed in the inactivated vaccine group. However, these findings should be interpreted cautiously because they may reflect differences in acute clinical presentation, inflammatory burden, or other unmeasured factors. It is known that thrombotic events after COVID-19 vaccination have been reported, albeit rarely, in a noteworthy manner [34,35]. The bidirectional interaction between inflammation and coagulation is explained within the framework of the concept of “immunothrombosis” [36]. Because the study was not designed to investigate vaccine-related thrombotic mechanisms, and because important indicators of NSTEMI severity differed between groups, these findings should not be interpreted as evidence of a specific vaccine-associated coagulation pathway. Previous studies have proposed several hypothetical mechanisms that could potentially influence inflammatory and coagulation pathways after vaccination [37,38]. However, none of these mechanisms were evaluated in the present study, and therefore they should not be interpreted as explanations for the observed biomarker differences.

Importantly, several clinical and laboratory variables differed significantly between the study groups, including blood pressure, body temperature, white blood cell count, neutrophil count, glucose, troponin, fibrinogen, and D-dimer levels. Therefore, differences observed in troponin and inflammatory markers may reflect variation in NSTEMI severity, infarct burden, timing of presentation, or other unmeasured clinical factors rather than vaccination-related biological effects. In addition, variables such as time from symptom onset to blood sampling, infarct size, culprit lesion characteristics, and acute treatment strategies were not systematically evaluated. Consequently, mechanistic interpretations should be considered hypothesis-generating rather than directly supported by the present data. Accordingly, these findings should be interpreted as descriptive observations from an exploratory observational study and should not be considered evidence of vaccine-specific biological effects or causal relationships. Although no significant differences in Galectin-3 levels were observed between vaccination groups, the possibility of residual confounding cannot be excluded. Because multivariable analyses adjusting for markers of NSTEMI severity were not performed, it remains possible that unmeasured differences in disease severity influenced the observed biomarker profiles.

The absence of a statistically significant difference in Galectin-3 levels between the groups in our study is consistent with the literature indicating that this biomarker is more closely associated with chronic inflammation and fibrotic remodeling processes [39,40]. It has been reported that Galectin-3, particularly in the post-infarction period, increases at a later stage in relation to myocardial fibrosis and remodeling processes and carries prognostic value [40,41]. Therefore, while necrosis markers such as troponin are expected to predominate in the acute phase of ACS, the lack of a significant early change in Galectin-3 levels is consistent with previous reports suggesting that this biomarker is more closely related to fibrotic remodeling processes than acute myocardial necrosis. In our study, the exclusion of individuals who received their last COVID-19 1 January 2022, was not intended to definitively claim the presence of a 3-year persistent chronic inflammation. Rather, this temporal criterion was methodologically applied to minimize the potential confounding effects of short-term, acute post-vaccination immune responses. By focusing exclusively on individuals with a more remote vaccination history, we aimed to perform an exploratory assessment of Galectin-3 levels without the interference of recent vaccine administration. Therefore, the single Galectin-3 measurement obtained at admission should be interpreted as a cross-sectional snapshot of patients with a historical vaccination background, rather than a definitive marker of long-term fibrotic burden. The main purpose of measuring Galectin-3 levels in the acute period in our study was to evaluate the possible effect of a post-vaccination chronic inflammatory background on acute coronary events. Although Galectin-3 is associated with inflammation, fibrosis, and tissue remodeling, the biological duration over which vaccination-related effects, if present, might influence Galectin-3 levels remains uncertain. Therefore, the present study should not be interpreted as an assessment of persistent vaccine-induced inflammation. Rather, it represents an exploratory evaluation of the relationship between vaccination history and Galectin-3 levels among patients presenting with NSTEMI. However, considering the temporal kinetics of this biomarker, the absence of a significant difference between the groups in the acute phase can be regarded as an understandable finding. In addition, within the scope of our study, Galectin-3 levels measured during the acute presentation of NSTEMI demonstrated that there was no statistically significant difference between vaccinated and unvaccinated groups in terms of cardiac injury and remodeling processes. However, given the observational cross-sectional design of the study and the single-time-point measurement of Galectin-3 during acute NSTEMI presentation, these findings should be interpreted as associations rather than evidence of causality. In the present study, Galectin-3 was measured at a single time point during the acute presentation of NSTEMI. Therefore, the measured values should be interpreted as reflecting the biological status present at hospital admission rather than dynamic changes over time. Because serial measurements were not available, temporal trajectories, post-infarction remodeling processes, and persistence of biomarker alterations could not be evaluated. Although Galectin-3 levels were numerically higher among participants with a history of COVID-19 infection, no statistically significant association was observed. However, this finding should be interpreted cautiously. Previous SARS-CoV-2 infection may influence inflammatory activity, endothelial function, thrombotic pathways, and cardiovascular risk, and the present study may have been underpowered to detect subtle differences related to infection history. Given the limited sample size, the unadjusted nature of the subgroup analysis, and the known effects of prior SARS-CoV-2 infection on inflammatory and thrombotic pathways, these findings should be interpreted cautiously. A potential confounding effect of prior COVID-19 infection cannot be excluded. The absence of detailed vaccination exposure data further limits interpretation of potential associations between vaccination history and Galectin-3 levels. Different vaccination schedules, booster doses, and intervals since vaccination may have distinct biological effects that could not be evaluated in the present study.

In our study, the similarity of Galectin-3 levels across groups may provide preliminary information regarding the relationship between vaccination history and biomarkers associated with inflammatory remodeling. Although several biological mechanisms have been proposed to explain potential cardiovascular effects of different COVID-19 vaccine platforms, including endothelial activation, inflammatory signaling, and coagulation-related pathways, the present study was not designed to directly evaluate these mechanisms. Importantly, none of these biological pathways were directly evaluated in the present study. Therefore, any discussion of such mechanisms should be interpreted as theoretical context derived from previous literature rather than evidence generated by the current data. Therefore, such explanations should be considered theoretical background rather than findings derived from the current data. Several hypothetical biological mechanisms have been proposed to explain potential cardiovascular effects following mRNA vaccination, including endothelial activation, inflammatory signaling, ACE2 modulation, and microvascular alterations [31,32,42]. However, none of these mechanisms were investigated in the present study. Therefore, they should be interpreted solely as theoretical background derived from previous literature rather than as mechanistic explanations for the findings observed in our cohort [19].

The biological effects of inactivated vaccines have also been discussed in the literature, and several hypotheses involving adjuvant-related immune activation and inflammatory responses have been proposed [43,44]. However, whether these proposed mechanisms translate into measurable differences in chronic inflammatory biomarkers, including Galectin-3, remains uncertain and was not investigated in the present study [19,45].

In conclusion, although several biological mechanisms have been proposed for different COVID-19 vaccine platforms, these pathways were not directly evaluated in the present study. Therefore, the absence of significant differences in Galectin-3 levels should not be interpreted as supporting or refuting any specific biological mechanism but rather as an observational finding within this study population [19,45]. However, the present study was not designed to evaluate long-term structural cardiac outcomes, and therefore such conclusions cannot be drawn from these findings [3].

Several alternative explanations should be considered when interpreting the present findings. First, the relatively small sample size may have limited the ability to detect subtle differences in Galectin-3 levels between groups. Second, residual confounding related to baseline clinical differences and prior COVID-19 infection cannot be excluded. Third, Galectin-3 was measured during the acute presentation of NSTEMI, and the kinetics of this biomarker may not fully reflect chronic inflammatory or fibrotic processes at a single time point. Finally, it is possible that Galectin-3 may not be sufficiently sensitive to detect subtle biological differences associated with vaccination history, if such differences exist.

### Limitations

Several limitations should be considered when interpreting the results of our study. Most importantly, Galectin-3 was measured only once during the acute presentation of NSTEMI. Therefore, the present findings should be interpreted as a cross-sectional assessment of biomarker levels at the time of admission rather than evidence regarding long-term inflammatory remodeling, persistent fibrosis, or chronic biological changes. Furthermore, mechanistic pathways such as endothelial dysfunction, ACE2 modulation, macrophage–fibroblast signaling, and collagen remodeling were not directly assessed and therefore cannot be inferred from the present findings. In addition, the absence of serial follow-up measurements precludes assessment of temporal trends, longitudinal remodeling processes, and the persistence of any observed biomarker patterns. Furthermore, the biological persistence of potential vaccination-related effects on Galectin-3 levels over several years remains uncertain and was not directly evaluated in the present study. First, the absence of a similar study in the literature, while increasing the originality of our work, limits the comparability of the findings. In addition, the relatively small sample size of 75 patients represents an important limitation. The single-center and cross-sectional design of the study also restricts the generalizability of the results to larger and more heterogeneous populations. Furthermore, Galectin-3 measurement was performed at a single time point, which did not allow the evaluation of the temporal changes in this biomarker. Furthermore, detailed indicators of NSTEMI severity, including time from symptom onset to blood sampling, infarct size, culprit lesion characteristics, and acute treatment variables, were not systematically collected. Therefore, the potential contribution of disease severity to differences in troponin and inflammatory markers cannot be excluded.

Prior COVID-19 infection represents a potential source of residual confounding because of its established effects on systemic inflammation, endothelial function, thrombosis, and cardiovascular outcomes. Although a subgroup analysis according to COVID-19 history was performed, the relatively small number of previously infected participants and the absence of multivariable adjustment limit definitive interpretation of these findings.

Detailed vaccination exposure characteristics, including the number of vaccine doses received, exact timing of the last vaccine dose, interval between vaccination and NSTEMI presentation, homologous versus heterologous vaccination schedules, and potential vaccine combinations, were not systematically available. Therefore, the relationship between vaccination exposure intensity and Galectin-3 levels could not be evaluated.

In addition, the exclusion of individuals with more recent vaccination exposure may have introduced selection bias and may limit the representativeness of the study population. Therefore, the findings may not be generalizable to patients with NSTEMI who received COVID-19 vaccination more recently.

In addition, multivariable analyses adjusting for potential indicators of NSTEMI severity, such as troponin levels, neutrophil count, inflammatory markers, and other baseline clinical differences, were not performed. Therefore, residual confounding related to differences in disease severity cannot be excluded and may have influenced the observed associations.

Although a subgroup analysis according to prior COVID-19 infection was performed, the relatively limited sample size may have reduced the ability to detect modest differences in Galectin-3 levels related to infection history. Therefore, residual confounding associated with previous SARS-CoV-2 infection cannot be excluded.

In addition, the exclusion of patients receiving statins and angiotensin-converting enzyme inhibitors was intended to minimize potential pharmacological influences on Galectin-3 levels. However, this approach resulted in a highly selected study population that may not fully reflect routine clinical practice. Because patients with NSTEMI commonly receive statins, ACE inhibitors/angiotensin receptor blockers, beta-blockers, antiplatelet agents, and other cardiovascular therapies, the generalizability of the present findings to broader NSTEMI populations may be limited.

In addition, recruitment was performed to achieve predefined group sizes rather than to reflect the natural distribution of vaccination status among patients presenting with NSTEMI. Consequently, the study population may not fully represent real-world vaccination patterns, which may further limit the generalizability of the findings.

Baseline differences in several vital signs and laboratory parameters among the study groups may have introduced potential confounding effects. However, due to the limited number of patients in each group, a reliable multivariable analysis including these variables could not be performed. This represents an important limitation of the study, and therefore, the findings should be interpreted as a preliminary evaluation of the association between different COVID-19 vaccine platforms and serum Galectin-3 levels in patients with NSTEMI, rather than as evidence of a causal relationship.

Taken together, several important methodological limitations should be considered when interpreting the present findings. These include the relatively small sample size, single-center design, cross-sectional study structure, lack of longitudinal follow-up, single-time-point measurement of Galectin-3, incomplete characterization of vaccination exposure, absence of robust multivariable adjustment, potential residual confounding related to NSTEMI severity and prior COVID-19 infection, and the limited representativeness of the study cohort resulting from the exclusion criteria. Accordingly, the findings should be regarded as exploratory and hypothesis-generating rather than definitive evidence regarding the relationship between COVID-19 vaccination history and Galectin-3 levels in patients with NSTEMI.

## 5. Conclusions

This study evaluated serum Galectin-3 levels in patients with angiographically confirmed ACS and found no statistically significant association between COVID-19 vaccination status and biomarker levels. The similarity of Galectin-3 levels across vaccinated and unvaccinated groups, a central indicator of inflammation, thrombosis, and fibrotic processes, clinically suggests that no significant association was observed between vaccination status and serum Galectin-3 levels in this cohort of NSTEMI patients. The observed increases in Troponin I and inflammatory cell parameters (WBC, neutrophils) may be related to differences in the severity of clinical presentation and other baseline characteristics among subgroups rather than reflecting vaccine-specific effects. In addition, the mild elevations in coagulation parameters (D-dimer, fibrinogen) observed in the inactivated vaccine group do not align with classical immune-thrombotic conditions, as they were not accompanied by platelet consumption.

In this exploratory and descriptive study of patients with NSTEMI, no statistically significant differences in serum Galectin-3 levels were observed according to COVID-19 vaccination status or vaccine type. These findings suggest that vaccination history was not associated with measurable differences in Galectin-3, a biomarker linked to inflammatory remodeling and fibrosis, within the study population. Although this observation does not permit causal inferences regarding long-term cardiovascular effects, it does not support the presence of a marked adverse chronic inflammatory signal related to prior COVID-19 vaccination. Further prospective studies with larger cohorts and longitudinal follow-up are required to clarify the relationship between vaccination history, inflammatory biomarkers, and cardiovascular outcomes.

## Figures and Tables

**Table 1:** Comparison of Galectin-3 levels, admission vital signs at presentation, and laboratory parameters according to groups.

Parameters	Group A (n = 25)	Group B (n = 25)	Group C (n = 25)	*p* Value
Age	65.72 ± 13.82	64.80 ± 10.71	62.92 ± 6.10	0.556 ^‡^
Initial Vital Signs				
SBP mmHg	130.00 (115.00–140.00) ^a.b^	120.00 (115.00–155.00) ^b^	120.00 (110.00–120.00) ^a^	0.022 ^†^
DBP mmHg	80.00 (70.00–85.00) ^a.b^	80.00 (80.00–90.00) ^b^	80.00 (70.00–80.00) ^a^	0.021 ^†^
HR/min	85.00 (76.50–92.00)	82.00 (74.50–90.00)	80.00 (75.00–85.00)	0.653 ^†^
Fever °C	36.60 (36.20–36.70) ^b^	36.50 (36.00–36.60) ^a.b^	36.20 (36.00–36.45) ^a^	0.006 ^†^
Initial Laboratory Results				
WBC	10.87 (6.93–14.15) ^b^	10.30 (8.27–12.20) ^b^	7.60 (6.16–8.51) ^a^	0.003 ^†^
PLT	224.00 (171.50–325.50)	248.00 (211.00–295.50)	231.00 (206.50–280.50)	0.934 ^†^
Neu	7.31 (4.07–11.31) ^b^	7.17 (5.03–8.28) ^b^	4.15 (3.43–5.42) ^a^	<0.001 ^†^
Lym	1.88 ± 1.19	2.19 ± 1.02	2.33 ± 0.73	0.295 ^‡^
Glu	142.00 (115.00–211.50) ^b^	149.00 (120.00–228.50) ^b^	94.00 (88.50–116.50) ^a^	<0.001 ^†^
IG Count	0.03 (0.02–0.08) ^b^	0.04 (0.03–0.06) ^b^	0.02 (0.01–0.03) ^a^	0.002 ^†^
Troponin	1.00 (0.43–2.90) ^b^	2.60 (0.62–9.43) ^b^	0.10 (0.10–0.72) ^a^	<0.001 ^†^
Fibrinogen	354.00 (273.50–396.00) ^a^	328.00 (292.50–363.50) ^a^	298.00 (235.00–351.00) ^a^	0.048 ^†^
D-Dimer	0.58 (0.33–1.10) ^b^	0.35 (0.21–0.57) ^a.b^	0.15 (0.10–0.35) ^a^	0.002 ^†^
Sodium	140.00 (137.50–141.00)	139.00 (136.00–141.00)	140.00 (139.00–141.00)	0.591 ^†^
Potassium	4.23 ± 0.49	4.26 ± 0.50	4.28 ± 0.44	0.629 ^‡^
Galectin-3 (ng/mL)	0.128 (0.102–0.166)	0.128(0.110–0.202)	0.120 (0.087–0.138)	0.481 ^†^

^†^ Kruskal–Wallis test. ^‡^ One-way ANOVA. Note: Continuous variables are presented as median (Q1–Q3). The Kruskal–Wallis test was used for comparisons between groups. Letter coding: For parameters showing significance in the overall comparison, pairwise comparisons were evaluated using the Dunn–Bonferroni test. There is no statistical difference between groups sharing the same letter at the Bonferroni-corrected post hoc *p* < 0.05 level. There is a statistically significant difference between groups bearing different letters. The code “a.b” indicates that the respective group is statistically similar to both “^a^” and “^b^”. Abbreviations: DBP: diastolic blood pressure; Glu: glucose; HR: heart rate; IG Count: immature granulocyte count; Lym: lymphocyte; Min: minute; Neu: neutrophil; PLT: platelet; SBP: systolic blood pressure; WBC: white blood cell count.

**Table 2:** Analysis of Clinical and Laboratory Findings According to History of COVID-19.

Parameters	History of COVID-19		*p*-Value
	No (n = 55)	Yes (n = 20)	
Age	62.50 (58–69)	68 (57–70)	0.621 ^†^
SBP	120 (110–133.75)	120 (110–140)	0.761 ^†^
DBP	80 (70–80)	80 (70–80)	0.506 ^†^
HR	80 (76–88.75)	85 (70–88)	0.956 ^†^
Fever	36.3 (36–36.5)	36.5 (36.2–36.7)	0.035 ^†^
WBC	8.57 (7.18–10.99)	9.71 (6.78–13.4)	0.394 ^†^
Platelet count	234 (205–286.75)	245 (195–296)	0.884 ^†^
Neutrophils	5.415 (4.01–7.69)	7.17 (3.89–10.58)	0.454 ^†^
Lymphocyte	2.15 ± 0.97	2.07 ± 1.13	0.759 ^‡^
Glucose	127 (94.25–215)	122 (107–148)	0.639 ^†^
IG Count	0.03 (0.02–0.05)	0.03 (0.02–0.09)	0.411 ^†^
Troponin	1 (0.1–3.76)	0.87 (0.31–7.45)	0.516 ^†^
Fibrinogen	310 (270–372)	340 (298–369)	0.511 ^†^
D-Dimer	0.295 (0.15–0.75)	0.38 (0.19–0.81)	0.783 ^†^
Sodium	139 (137–141)	140 (138–142)	0.199 ^†^
Potassium	4.26 (4.02–4.55)	4.1 (3.77–4.54)	0.120 ^†^
Galectin-3 (ng/mL)	0.126 (0.096–0.155)	0.134 (0.104–0.219)	0.320 ^†^

^†^ Mann–Whitney U Test. ^‡^ Independent Samples *t*-test. COVID-19: Coronavirus disease 2019; DBP: Diastolic blood pressure; HR: Heart rate; IG: Immature granulocyte. SBP: Systolic blood pressure; WBC: White blood cell count.

**Table 3:** Dunn-Bonferroni post hoc pairwise comparison results.

Parameters	Pairwise Comparison	Test Statistic	Standard Error	Standard Test Statistic	*p*	Adjusted *p*-Value
SBP	Group C—Group A	14.480	6.045	2.395	0.017	0.050
SBP	Group C—Group B	14.500	6.045	2.399	0.016	0.049
SBP	Group A—Group B	−0.020	6.045	−0.003	0.997	1.000
DBP	Group C—Group A	7.620	5.811	1.311	0.190	0.569
DBP	Group C—Group B	16.140	5.811	2.778	0.005	0.016
DBP	Group A—Group B	−8.520	5.811	−1.466	0.143	0.428
Fever	Group C—Group B	9.080	6.052	1.500	0.134	0.401
Fever	Group C—Group A	19.480	6.052	3.219	0.001	0.004
Fever	Group B—Group A	10.400	6.052	1.719	0.086	0.257
WBC	Group C—Group A	17.120	6.164	2.777	0.005	0.016
WBC	Group C—Group B	19.120	6.164	3.102	0.002	0.006
WBC	Group A—Group B	−2.000	6.164	−0.324	0.746	1.000
Neu	Group C—Group A	19.800	6.164	3.212	0.001	0.004
Neu	Group C—Group B	21.480	6.164	3.485	<0.001	0.001
Neu	Group A—Group B	−1.680	6.164	−0.273	0.785	1.000
Glu	Group C—Group A	21.920	6.163	3.557	<0.001	0.001
Glu	Group C—Group B	26.140	6.163	4.242	<0.001	<0.001
Glu	Group A—Group B	−4.220	6.163	−0.685	0.494	1.000
IG Count	Group C—Group A	15.660	6.071	2.579	0.010	0.030
IG Count	Group C—Group B	20.160	6.071	3.321	<0.001	0.003
IG Count	Group A—Group B	−4.500	6.071	−0.741	0.459	1.000
Troponin	Group C—Group A	19.700	6.128	3.215	0.001	0.004
Troponin	Group C—Group B	27.940	6.128	4.559	<0.001	<0.001
Troponin	Group A—Group B	−8.240	6.128	−1.345	0.179	0.536
Fibrinogen	Group C—Group B	11.100	6.164	1.801	0.072	0.215
Fibrinogen	Group C—Group A	14.520	6.164	2.356	0.018	0.055
Fibrinogen	Group B—Group A	3.420	6.164	0.555	0.579	1.000
D-Dimer	Group C—Group B	12.660	6.147	2.060	0.039	0.118
D-Dimer	Group-C—Group A	21.600	6.147	3.514	<0.001	0.001
D-Dimer	Group B—Group A	8.940	6.147	1.454	0.146	0.437
Galectin-3	Group C—Group A	5.020	6.160	0.815	0.415	1.000
Galectin-3	Group C—Group B	7.280	6.160	1.182	0.237	0.712
Galectin-3	Group A—Group B	−2.260	6.160	−0.367	0.714	1.000

Note: Each row tests the null hypothesis that the distributions of the respective two groups are identical. Multiple comparison correction was performed using the Bonferroni method. Abbreviations: DBP: diastolic blood pressure; Glu: glucose; IG Count: immature granulocyte count; Neu: neutrophil; SBP: systolic blood pressure; SE: standard error; WBC: white blood cell count.

**Table 4:** General linear models for log-transformed Galectin-3 levels in the overall cohort.

Variable	Model 1 B (95% CI), *p*	Model 2 B (95% CI), *p*	Model 3 B (95% CI), *p*
Group A vs. Group C	−0.001 (−0.403 to 0.400), *p* = 0.995	−0.018 (−0.434 to 0.398), *p* = 0.930	0.270 (−0.260 to 0.800), *p* = 0.313
Group B vs. Group C	0.065 (−0.336 to 0.467), *p* = 0.747	0.045 (−0.366 to 0.456), *p* = 0.828	0.247 (−0.272 to 0.765), *p* = 0.345
Female vs. Male	—	−0.317 (−0.654 to 0.019), *p* = 0.064	−0.298 (−0.665 to 0.069), *p* = 0.110
No prior COVID-19 vs. prior COVID-19	—	−0.090 (−0.482 to 0.301), *p* = 0.647	−0.150 (−0.587 to 0.286), *p* = 0.493
Age	—	0.006 (−0.010 to 0.022), *p* = 0.442	0.004 (−0.013 to 0.021), *p* = 0.654
SBP	—	—	−0.001 (−0.008 to 0.007), *p* = 0.837
Fever	—	—	−0.527 (−1.141 to 0.087), *p* = 0.091
WBC	—	—	−0.043 (−0.102 to 0.016), *p* = 0.153
Glucose	—	—	−0.00008 (−0.003 to 0.003), *p* = 0.959
Troponin	—	—	0.005 (−0.018 to 0.028), *p* = 0.652
Fibrinogen	—	—	−0.001 (−0.003 to 0.002), *p* = 0.675
D-dimer	—	—	0.028 (−0.182 to 0.237), *p* = 0.793
Overall group effect	F = 0.072, *p* = 0.931	F = 0.053, *p* = 0.949	F = 0.583, *p* = 0.562
R^2^/Adjusted R^2^	0.002/−0.026	0.056/−0.012	0.124/−0.045
Levene’s test	*p* = 0.730	*p* = 0.271	*p* = 0.414

Note: The dependent variable was natural log-transformed Galectin-3. The unvaccinated NSTEMI group, male sex, and previous COVID-19 history were used as reference categories. B coefficients represent the difference in Ln(Galectin-3) compared with the reference category or the expected change per unit increase for continuous covariates. Levene’s test was used to evaluate homogeneity of error variances. Model 1 included vaccination group only. Model 2 was adjusted for age, sex, and previous COVID-19 history. Model 3 was additionally adjusted for systolic blood pressure, body temperature, WBC count, glucose, troponin, fibrinogen, and D-dimer. Abbreviations: B: regression coefficient; CI: confidence interval; COVID-19: coronavirus disease 2019; Ln: natural logarithm; NSTEMI: non-ST-segment elevation myocardial infarction; SBP: systolic blood pressure; WBC: white blood cell count.

**Table 5:** Estimated marginal means of log-transformed Galectin-3 levels in the overall cohort.

Group	Model 1 Mean Ln(Galectin-3), 95% CI	Model 2 Adjusted Mean Ln(Galectin-3), 95% CI	Model 3 Adjusted Mean Ln(Galectin-3), 95% CI
Group A	4.867 (4.583–5.150)	4.853 (4.564–5.141)	4.994 (4.670–5.318)
Group B	4.933 (4.649–5.217)	4.916 (4.624–5.208)	4.971 (4.659–5.283)
Group C	4.868 (4.584–5.152)	4.871 (4.542–5.200)	4.724 (4.326–5.123)
Overall group *p*	0.931	0.949	0.562

Note: Values are presented as estimated marginal means of Ln(Galectin-3) with 95% confidence intervals. Model 1 included vaccination group only. Model 2 was adjusted for age, sex, and previous COVID-19 history. Model 3 was additionally adjusted for systolic blood pressure, body temperature, WBC count, glucose, troponin, fibrinogen, and D-dimer. Bonferroni-adjusted pairwise comparisons were non-significant in all models. Abbreviations: CI: confidence interval; COVID-19: coronavirus disease 2019; Ln: natural logarithm; SBP: systolic blood pressure; WBC: white blood cell count.

**Table 6:** General linear models for log-transformed Galectin-3 levels after excluding patients with previous COVID-19 infection.

Variable	Model 1 B (95% CI), *p*	Model 2 B (95% CI), *p*	Model 3 B (95% CI), *p*
Group A vs. Group C	−0.217 (−0.619 to 0.186), *p* = 0.285	−0.198 (−0.593 to 0.197), *p* = 0.319	0.020 (−0.502 to 0.541), *p* = 0.940
Group B vs. Group C	0.128 (−0.260 to 0.516), *p* = 0.511	0.151 (−0.230 to 0.532), *p* = 0.430	0.333 (−0.181 to 0.847), *p* = 0.198
Female vs. Male	—	−0.336 (−0.673 to 0.000), *p* = 0.050	−0.243 (−0.619 to 0.132), *p* = 0.198
Age	—	−0.001 (−0.018 to 0.016), *p* = 0.901	0.002 (−0.016 to 0.021), *p* = 0.800
SBP	—	—	0.002 (−0.007 to 0.010), *p* = 0.669
Fever	—	—	−0.554 (−1.156 to 0.047), *p* = 0.070
WBC	—	—	0.016 (−0.052 to 0.085), *p* = 0.629
Glucose	—	—	−0.001 (−0.004 to 0.002), *p* = 0.433
Troponin	—	—	0.003 (−0.018 to 0.025), *p* = 0.750
Fibrinogen	—	—	−0.002 (−0.005 to −0.00003), *p* = 0.048
D-dimer	—	—	0.025 (−0.156 to 0.206), *p* = 0.782
Overall group effect	F = 1.315, *p* = 0.277	F = 1.390, *p* = 0.259	F = 1.310, *p* = 0.280
R^2^/Adjusted R^2^	0.048/0.012	0.123/0.053	0.266/0.079
Levene’s test	*p* = 0.838	*p* = 0.298	*p* = 0.297

Note: This sensitivity analysis was performed after excluding patients with previous COVID-19 infection. The dependent variable was natural log-transformed Galectin-3. Model 1 included vaccination group only. Model 2 was adjusted for age and sex. Model 3 was additionally adjusted for systolic blood pressure, body temperature, WBC count, glucose, troponin, fibrinogen, and D-dimer. The unvaccinated NSTEMI group and male sex were used as reference categories. B coefficients represent the difference in Ln(Galectin-3) compared with the reference category or the expected change per unit increase for continuous covariates. Levene’s test was used to evaluate homogeneity of error variances. Abbreviations: B: regression coefficient; CI: confidence interval; COVID-19: coronavirus disease 2019; Ln: natural logarithm; NSTEMI: non-ST-segment elevation myocardial infarction; SBP: systolic blood pressure; WBC: white blood cell count.

**Table 7:** Estimated marginal means of log-transformed Galectin-3 levels after excluding patients with previous COVID-19 infection.

Group	Model 1 Mean, 95% CI	Model 2 Adjusted Mean, 95% CI	Model 3 Adjusted Mean, 95% CI
Group A	4.645 (4.331–4.958)	4.611 (4.302–4.920)	4.724 (4.380–5.068)
Group B	4.989 (4.695–5.283)	4.960 (4.670–5.251)	5.038 (4.710–5.366)
Group C	4.861 (4.608–5.114)	4.809 (4.556–5.062)	4.705 (4.381–5.028)
Overall group *p*	0.277	0.259	0.280

Note: Values are presented as estimated marginal means of Ln(Galectin-3) with 95% confidence intervals after excluding patients with previous COVID-19 infection. Model 1 included vaccination group only. Model 2 was adjusted for age and sex. Model 3 was additionally adjusted for systolic blood pressure, body temperature, WBC count, glucose, troponin, fibrinogen, and D-dimer. Bonferroni-adjusted pairwise comparisons were non-significant in all models. Abbreviations: CI: confidence interval; COVID-19: coronavirus disease 2019; Ln: natural logarithm; SBP: systolic blood pressure; WBC: white blood cell count.

## Data Availability

The raw data supporting the conclusions of this article will be made available by the authors on request.

## Abbreviations

The following abbreviations are used in this manuscript:

ACS	Acute Coronary Syndrome
ACE2	Angiotensin-Converting Enzyme 2
BNT162b2	Pfizer–BioNTech COVID-19 mRNA Vaccine
COVID-19	Coronavirus Disease 2019
DBP	Diastolic Blood Pressure
D.U.A.	Diler Us Altay
ELISA	Enzyme-Linked Immunosorbent Assay
EMA	European Medicines Agency
ESC	European Society of Cardiology
HR	Heart Rate
IGG	Immature Granulocyte Count
NSTEMI	Non-ST-Segment Elevation Myocardial Infarction
SBP	Systolic Blood Pressure
SpO_2_	Peripheral Oxygen Saturation
WBC	White Blood Cell Count
